# Nailfold capillaroscopy and candidate-biomarker levels in systemic sclerosis-associated pulmonary hypertension: A cross-sectional study

**DOI:** 10.1177/23971983231175213

**Published:** 2023-05-22

**Authors:** Jacqueline MJ Lemmers, Arjan PM van Caam, Brigit Kersten, Cornelia HM van den Ende, Hanneke Knaapen, Arie PJ van Dijk, Wanda Hagmolen of ten Have, Frank HJ van den Hoogen, Hans Koenen, Sander I van Leuven, Wynand Alkema, Ruben L Smeets, Madelon C Vonk

**Affiliations:** 1 Department of Rheumatology, Radboud University Medical Center, Nijmegen, The Netherlands; 2 Laboratory of Experimental Rheumatology, Radboud University Medical Center, Nijmegen, The Netherlands; 3 Department of Cardiology, Radboud University Medical Center, Nijmegen, The Netherlands; 4 Department of Pulmonary Diseases, Radboud University Medical Center, Nijmegen, The Netherlands; 5 Laboratory of Clinical Chemistry and Immunology, Radboud University Medical Center, Nijmegen, The Netherlands; 6 Department of Data Science for Life Sciences & Health, Hanze University, Groningen, The Netherlands

**Keywords:** Systemic sclerosis, pulmonary hypertension, nailfold capillaroscopy, candidate-biomarkers, pulmonary arterial hypertension

## Abstract

**Objectives:**

Pulmonary hypertension is one of the leading causes of death in systemic sclerosis. Early detection and treatment of pulmonary hypertension in systemic sclerosis is crucial. Nailfold capillaroscopy microscopy, vascular autoantibodies AT1R and ETAR, and several candidate-biomarkers have the potential to serve as noninvasive tools to identify systemic sclerosis patients at risk for developing pulmonary hypertension. Here, we explore the classifying potential of nailfold capillaroscopy microscopy characteristics and serum levels of selected candidate-biomarkers in a sample of systemic sclerosis patients with and without different forms of pulmonary hypertension.

**Methods:**

A total of 81 consecutive systemic sclerosis patients were included, 40 with systemic sclerosis pulmonary hypertension and 41 with no pulmonary hypertension. In each group, quantitative and qualitative nailfold capillaroscopy microscopy characteristics, vascular autoantibodies AT1R and ETAR, and serum levels of 24 soluble serum factors were determined. For evaluation of the nailfold capillaroscopy microscopy characteristics, linear regression analysis accounting for age, sex, and diffusing capacity of the lungs for carbon monoxide percentage predicted was used. Autoantibodies and soluble serum factor levels were compared using two-sample *t* test with equal variances.

**Results:**

No statistically significant differences were observed in quantitative or qualitative nailfold capillaroscopy microscopy characteristics, or vascular autoantibody ETAR and AT1R titer between systemic sclerosis–pulmonary hypertension and systemic sclerosis–no pulmonary hypertension. In contrast, several serum levels of soluble factors differed between groups: Endostatin, sVCAM, and VEGFD were increased, and CXCL4, sVEGFR2, and PDGF-AB/BB were decreased in systemic sclerosis–pulmonary hypertension. Random forest classification identified Endostatin and CXCL4 as the most predictive classifiers to distinguish systemic sclerosispulmonary hypertension from systemic sclerosis-no pulmonary hypertension.

**Conclusion:**

This study shows the potential for several soluble serum factors to distinguish systemic sclerosis-pulmonary hypertension from systemic sclerosis-no pulmonary hypertension. We found no classifying potential for qualitative or quantitative nailfold capillaroscopy microscopy characteristics, or vascular autoantibodies.

## Significance and innovation

### What this study adds

Systemic sclerosis patients with and without associated pulmonary hypertension can be differentiated based on the serum cytokine expression profile, in which elevated Endostatin in patients with associated pulmonary hypertension is the most promising discriminative factor in this study.

### Innovation

We show potential for a machine learning–based random forest model to be of aid in selecting the most discriminative markers in this study.

### How this study might affect research

This study serves as a basis for designing future longitudinal studies exploring if these factors are present before clinical diagnosis of pulmonary hypertension in systemic sclerosis, and explore if they could be of added value to current screenings algorithms and risk stratification for pulmonary arterial hypertension in systemic sclerosis.

## Introduction

Systemic sclerosis (SSc) is a severe autoimmune connective tissue disease characterized by microvascular dysfunction, inflammation, and autoimmune dysregulation, resulting in fibrosis of the skin and internal organs with a variable degree of severity. SSc causes organ damage, impaired quality of life, and increased mortality.^1^ Although the pathogenesis of SSc is largely unknown to date, it is hypothesized that there is an early insult affecting the microvasculature followed by an ongoing process resulting in diffuse vascular damage. Due to heterogeneity in the development of clinical complications, only a subset of patients will develop severe vascular events such as pulmonary hypertension (PH).^2^

PH is a lethal complication of SSc, and various clinical phenotypes may develop.^3^ In the majority of cases, PH in SSc is caused by progressive vasculopathy of the small pulmonary arteries, leading to pulmonary arterial hypertension (PAH, WHO group I). This occurs in 1%–2% of SSc patients per year and ultimately develops in 8%–12% of patients.^4–6^ Alternatively, one-third of PH cases in SSc is associated with cardiac disease (WHO group II) or severe pulmonary fibrosis with hypoxia (WHO group III).^7^ Many patients with SSc-associated PH (SSc-PH) suffer from combinations of two or more phenotypes of PH. As vasoactive therapy is only indicated in PAH, expert assessment is crucial to evaluate what is the main contributing form of PH and to decide which patients are eligible for vasoactive treatment.^3^

Despite targeted treatment for PAH, 3-year survival after diagnosis is estimated to be only 56%.^8^ Early diagnosis and treatment is essential, as this results in better outcomes, decreasing both morbidity and mortality.^9^ In the past decade, several screening-guidelines and risk assessment algorithms have been developed to aid the early detection of established SSc-PAH, such as the DETECT algorithm and the regularly updated ECS/ERS guidelines.^10,11^ However, the optimal frequency of screening is unknown to date. If patients with SSc at high risk for development of P(A)H could be identified, screening modalities could be tailored based on this risk profile, making early treatment possible.^12^

As vasculopathy occurs early in the SSc disease process, noninvasive markers assessing microvascular function are good candidates for risk stratification. In SSc, vasculopathy results in early impaired capillary function by loss of architecture and loss of capillaries.^13^ These specific SSc features can be visualized by nailfold capillary microscopy (NCM), a noninvasive technique which is currently used to aid in SSc diagnosis.^14^ Previous studies have described a possible association of loss of capillary density and a “late” scleroderma pattern with SSc-PAH or organ involvement.^14–22^ However, these data on NCM parameters and patterns associated with SSc-PAH are limited, outcomes are contradictive, and most studies are limited due to a low number of patients. Furthermore, data on NCM pattern associated with other forms of PH in SSc are lacking.

Next to NCM, several soluble serum factors such as the vascular autoantibodies anti-endothelin-1 type A receptor antibody (anti-ETAR) and anti-angiotensin II type1- receptor antibody (antiAT1R) were identified as potential biomarkers for the development of complications such as pulmonary fibrosis, digital ulcers (DUs), and SSc-PAH.^23,24^ Also, several cytokines and chemokines reflecting angiogenesis, endothelial damage, fibrosis, or immunological imbalance have been suggested as potential biomarker candidates for SSc-PAH.^25–32^ Our research group recently suggested that moving from single biomarkers toward pathophysiological panels consisting of multiple biomarkers should be the way forward in risk stratification of SSc patients.^33^

Therefore, in this study we investigated several noninvasive candidate-biomarkers and their potential to discriminate SSc-PH patients from SSc-noPH patients. Our hypothesis was that patients with SSc-PH display (1) a more advanced disturbed capillary image/capillary architecture reflected in a late capillary NCM pattern and/or capillary loss, (2) increased vascular autoantibodies, and (3) up- or downregulation of selective factors reflecting angiogenesis, endothelium damage, and vasculogenesis.

## Method

This study was performed at the Radboud University Medical Center, Nijmegen, the Netherlands, which is a tertiary referral center for SSc and PH center of excellence. All patients with a diagnosis of SSc-PH that visited the outpatient clinic during 1 year (02-2018 until 02-2019) were asked to participate. Furthermore, during this same period, consecutive SSc patients without PH were asked to serve as the control group. Although no formal power calculation was performed, we did take into account the following sample size considerations regarding capillary density: previous research in patients with SSc versus SSc-PAH has shown that a decreased capillary density was present in 42% versus 92% of the patients.^17^ Assuming that the proportion of patients with a decreased capillary density would be present in 40% of the SSc patients without SSc-PH, we hypothesized that a sample size of 30 patients per group would be sufficient to detect a difference of at least 30% between groups (one-sided tested, alpha = 0.05, 1 − beta = 0.80).

Inclusion criteria were (1) fulfillment of the 2013 European League Against Rheumatism criteria for SSc,^34^ (2) age > 18 years, and for the PH patients (3) PH diagnosed by right heart catheterization (RHC). We excluded SSc patients with overlap syndromes other than secondary Sjogren Syndrome. Disease onset of SSc was defined as the date of clinical SSc diagnosis. All clinical parameters and medication use were collected from the patient electronic medical file at the date of NCM performance, laboratory measurements (GFR, urate, NTproBNP), results of pulmonary function tests (PFT), and 6-min-walking-distance (6MWD) performed for standard clinical evaluation were collected with a maximum range of 1 year around the inclusion date.

Clinical aspects of SSc such as subclassification, presence of Raynaud’s phenomenon (RP), telangiectasias, DU past or present, and WHO Functional Class (NYHA) were collected. The presence of interstitial lung disease (ILD) was determined by High-Resolution Computer Tomography (HRCT). PAH (WHO group I) was defined by the 2015 criteria, namely, as mean pulmonary arterial pressure (mPAP) ⩾ 25 mmHg with pulmonary artery wedge pressure (PAWP) ⩽ 15 mmHg, and pulmonary vascular resistance (PVR) > 3 Wood units.^11^ All other forms of PH associated with SSc, in our study presented as “other PH,” were defined as follows: PH secondary to cardiac disease (WHO group II), PH secondary to pulmonary disease (WHO group III), other WHO groups, or combination of groups.^11^

Images of nailfold capillaries were acquired using an optical probe video capillaroscope equipped with a 200× contact lens and connected to image analysis software (Optilia OP-120 011, Mediscope Digital, videomicroscope (USB interface), OptiPix Capillaroscopy software, clinic 1.7× with a 200× high-resolution objective lens with unpolarized light). Two images per finger were evaluated, which consisted of both quantitative and qualitative parameters, as described by the EULAR Study Group on Microcirculation in Rheumatic Diseases.^14^ Evaluation of images was performed by two experienced physicians (J.M.J.L. and B.K.), blinded for patient diagnosis. If the two physicians did not reach consensus, a third physician (M.C.V.) evaluated the images to reach consensus.

Peripheral blood samples of the included patients were drawn and centrifugated at 4200 G for 10 min, then aliquoted and stored at −80°C until further processing.

The vascular receptor antibodies AT1R and ETAR were measured in duplicate for each patient using validated enzyme-linked immunosorbent assay (ELISA; Celltrend GmbH, Luckenwalde, Germany).

We selected 24 candidate cytokines or chemokines possibly discriminative for PH in SSc based on the recent literature and commercial availability.^26^ Serum levels of IL4, IL6, IL8, IL13, PDGFAA, PDGFAB-BB, 6Ckine, sTRAIL, MMP1, MMP7, sICAM1, sVCAM, CCL19/MIP3b, Endostatin, sVEGFR1, sVEGFR2, sVEGFR3, CXCL4, Endothelin1, FGF1, FGF2, VEGF-A, VEGF-C, and VEGF-D were measured using Miliplex kits, (Luminex Technology, Merck KGaA, Darmstadt, Germany). For all cytokines and chemokines, the lower limits of detection are included in the Supplemental Table C. Levels below the lower limit of detection were replaced by the lowest measurable value, that is, −10% of the lower limit of the calibration graph.

Baseline characteristics were described by descriptive statistics. For assessment of NCM characteristics between the SSc-PH and SSc-noPH groups, linear regression analysis accounting for age, SSc disease duration, and DLCO% predicted was used. Candidate-biomarker levels were compared by two-sample *t*-test with equal variances. Statistical significance was set at *p* < 0.05. The results were calculated using the computer software STATA/SE 16.0.

To evaluate the use of the candidate-biomarkers to discriminate between SSc-PH and SSc-noPH patients, a classification model using random forest (RF) methodology was generated. First, the dataset was randomly split into a training set (70% of the samples) and a test-set (30% of the samples), stratified by the presence of SSc-PH. Second we developed an RF model in the training set, utilizing machine learning techniques. The outcome of this model is based on 500 decision trees to explore the predictive value of each biomarker. Finally, we constructed a receiver operating characteristic (ROC) curve based on the test-set, and calculated the area under the curve (AUC) of the model with the best fit. All data were analyzed using the statistical packages installed under R version 3.6.2 (https://www.r-project.org, accessed on 20 December 2021).

This study complied with the Declaration of Helsinki, has been approved by the local ethics committee, and evaluated not to fall within the remit of Medical Research Involving Human Subjects Act (WMO). This study was approved on the basis of the Dutch Code of conduct for health research, the Dutch Code of conduct for responsible use, the Dutch Personal Data Protection Act, and the Medical Treatment Agreement Act (Radboudumc, Nijmegen, The Netherlands, File number CMO: 2017-3979). All clinical data were coded and stored in the Castor Electronical Database. Written informed consent was obtained from all the participants.

## Results

### Patient population

This cross-sectional study consisted of 81 SSc patients, of whom 40 had SSc-PH. Of these, 21 had SSc-PAH, and 19 had another form of associated PH (SSc-PH other). In both SSc-PH and SSc-noPH groups the majority of patients, 75% and 71%, respectively, were female and most patients had a limited cutaneous subtype. In the SSc-noPH group, nine patients received PH-mono-therapy (Bosentan) indicated for treatment of DUs. The clinical characteristics are presented in Table 1, and Supplemental Table A.

**Table 1.** Clinical characteristics SSc duration (*y*) measured as from the date of diagnosis until inclusion. PAH duration (*y*) measured as from the date of diagnosis by right heart catheterization until inclusion. Values presented as *n* (%) or mean ± SD.

Clinical characteristics	SSc-PH (*n* = 40)	SSc-noPH (*n* = 41)
Age, years (mean ± SD)	70.8 (9.0)	59.49 (11.9)
Female (*n*, %)	30 (75%)	29 (71%)
SSc duration years (mean ± SD)	12.4 (10.3)	6.8 (5.4)
SSc subtype		
LcSSc (*n*, %)	32 (80%)	27 (66%)
DcSSc (*n*, %)	8 (20%)	14 (34%)
ILD (*n*%)	26 (65%)	18 (44%)
Digital ulcers (*n*, %)	21 (53%)	17 (42%)
Telangiectasias (*n*, %)	39 (98%)	37 (90%)
NYHA class (*n*, %)		
I/II	2 (5%)/10 (25%)	20 (49%)/8 (20%)
III/IV	21 (52%)/7 (18%)	1 (2%)/0
Not reported	0	12 (29%)
Creatinine µmol/L (mean ± SD)	102 (26)	74.4 (17)
Urate mmol/L (mean ± SD)	0.41 (0.14) *n* = 36	0.29 (0.09)
NTproBNP pg/mL (mean ± SD)	1382 (2096)	364 (798)
ANA positive (*n*, %)	36 (90%) *n* = 36	39 (95%)
ACA	20 (50%)	10 (25%)
Anti-topoisomerase I	4 (10%)	11 (28%)
antiRNAIII	1 (3%)	4 (10%)
Other	12 (30%)	7 (17.1)
VC%pred (mean ± SD)	86 (20) *n* = 35	93 (20) *n* = 37
DLCO%pred (mean ± SD)	38 (9) *n* = 34	63 (14) *n* = 39
Immunosuppression (*n* %)	21 (53%)	27 (66%)
PH medication (*n* %)		
No-PH medication	6 (15%)	32(78%)
Mono therapy	10 (25%)	9 (22%)
Duo therapy	16 (40%)	0
Triple therapy	8 (20%)	0

Abbreviations: ACA: anti-centromere antibodies; ANA: antinuclear antibodies; antiRNAIII: anti-RNA polymerase III antibodies; DcSSc: diffuse cutaneous systemic sclerosis; DLCO%pred: diffusion capacity for carbon monoxide as percentage of predicted; ILD: interstitial lung disease; LcSSc: limited cutaneous systemic sclerosis; NTproBNP: N-terminal pro hormone of brain natriuretic peptide; NYHA: New York heart association functional class; PH: pulmonary hypertension; SSc: systemic sclerosis; VC%pred; vital capacity as percentage of predicted.

### Nailfold capillaroscopic characteristics

**Figure 1.**
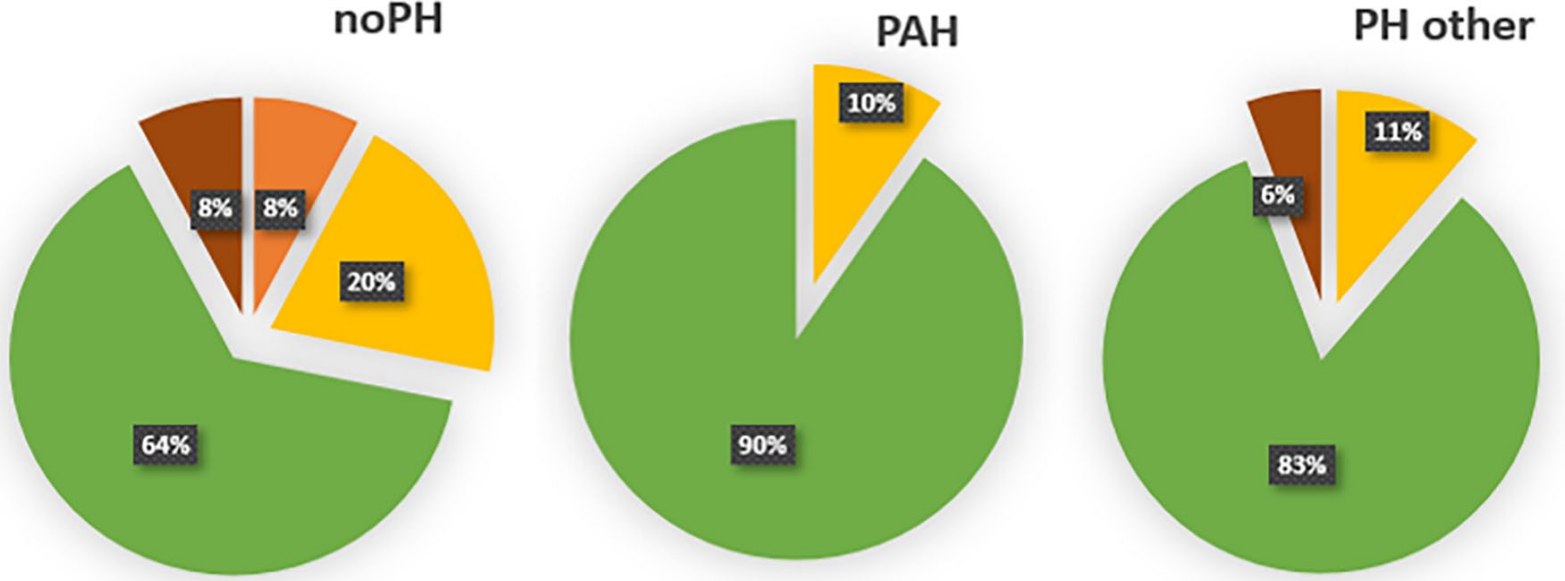
Qualitative nailfold capillaroscopic characteristics, presented per subgroup. noPH *n* = 39, PAH *n* = 21, PH-other *n* = 19. Colors: green is late scleroderma pattern, yellow is active scleroderma pattern, orange is early scleroderma pattern, and brown is non-scleroderma pattern. Abbreviations: PAH: pulmonary arterial hypertension; PH: pulmonary hypertension; SSc Systemic sclerosis.

We observed no significant differences in quantitative nailfold characteristics, accounting for age, SSc disease duration, and DLCO % predicted, between SSc patients with or without PH (Table 2). Furthermore, we did not observe significant differences between subgroups, comparing patients with associated PAH, other types of PH, or no-PH specifically (for additional information see Supplemental Table B). Although not statistically significant, distribution of overall NCM pattern seemed to differ between the subgroups. No early or non-scleroderma patterns were found in the SSc-PAH patients, whereas in SSc patients without PH all patterns were observed (Figure 1). For one SSc-noPH and one SSc-PH patient, NCM images were not interpretable due to low image quality, and in one SSc-noPH patient, NCM was not performed.

**Table 2.** Overview of quantitative nailfold capillaroscopic characteristics, values per group presented as mean (SD); results of linear regression analysis for each variable accounting for age, SSc duration and DLCO% predicted.

NCM	SSc PH (*n* = 40)	SSc noPH (*n* = 39)	*p* value	B-coefficient	95% CI
Capillary density/mm (SD)	5.0 (1.4)	5.4 (1.9)	0.76	0.20	−1.13 to 1.54
Digits with density ⩽ 3 (SD)	2.3 (1.9)	1.8 (2.1)	0.67	−0.35	−1.98 to 1.28
Giant capillaries/mm (SD)	0.16 (0.2)	0.2 (0.24)	0.27	−0.11	−0.31 to 0.09
Digits with hemorrhages (SD)	1.8 (1.9)	1.6 (1.5)	0.59	−0.39	−1.81 to 1.03
Abnormally shaped capillaries/mm (SD)	(0.47)	0.99 (0.55)	0.95	0.012	−0.38 to 0.40

Abbreviations: CI: confidence-interval; NCM: nailfold capillary microscopy; PH: pulmonary hypertension; SD: standard deviation; SSc: systemic sclerosis.

### Levels of vascular autoantibodies and cytokines

The levels of 26 soluble serum factors (including anti-ETAR and anti-AT1R) were analyzed, of which 6 factors were significantly different in SSc-PH patients; Endostatin, sVCAM, and VEGF-D were significantly increased, whereas levels of PF4/CXCL4, sVEGFR2, and PDGF-AB-BB were significantly decreased compared with SSc patients without PH.

Levels of all soluble factors are presented in Table 3. Exploring the distribution in the SSc-PAH subgroup, compared with SSc-noPH, the same factors remain significantly up- or downregulated, except for VEGFD, which shows no statistically significant elevation in SSc-PAH alone (data not shown).

**Table 3.** Overview of serum levels of all measured cytokines, chemokines and vascular autoantibodies.

Soluble serum factors	SSc-PH (*n* = 33) Mean (SD)	SSc-noPH (*n* = 41) Mean (SD)	*p* value	95% CI of difference between groups
Anti- AT1R (U/mL)	15.7 (8.7)	16.3 (9.5)	0.79	−0.58 [−4.8 to 3.7]
Anti-ETAR (U/mL)	14.1 (10.3)	14.4 (9.1)	0.88	−0.35 [−4.9 to 4.1]
CCL19 (pg/mL)	335 (149)	335 (170)	0.98	0.7 [−74 to 76]
CCL21 (6Ckine) (pg/mL)	101 (42)	82 (106)	0.65	19 [−63 to 102]
CXCL4 (ng/mL)*	3636 (1561.3)	5599 (2493)	**0.0002**	−1963 [−2956 to −970]
Endothelin1 (pg/mL)	14 (25)	9.0 (4)	0.16	5.6 [−2 to 14]
Endostatin (ng/mL)*	59 (21)	43 (25)	**0.004**	16 [5 to 27]
FGF1 (pg/mL)	9 (26)	3 (8)	0.23	5.1 [−3 to 14]
FGF2 (pg/mL)	145 (166)	118 (49)	0.33	27 [−28 to 81]
IL4 (pg/mL)	5 (6.8)	8 (26.3)	0.61	−2 [−12 to 7)
IL6 (pg/mL)	11 (26)	4.0 (4.0)	0.07	7 [−0.6 to 16]
IL8 (pg/mL)	12 (18)	8.0 (4.0)	0.14	4 [−1 to 10]
IL13 (pg/mL)	158 (191)	153 (164)	0.91	5.0 [−78 to 87]
MMP1 (pg/mL)	10,583 (6767)	11,061 (6048)	0.75	−478 [−3451 to 2496]
MMP7 (pg/mL)	12,538 (7443)	10,966 (7076)	0.36	1572 [−1804 to 4948]
PDGF-AA (pg/mL)	6440 (2264)	7222 (2443)	0.16	−782 [−1184 to 321]
PDGF AB-BB (pg/mL)*	29,386 (6339)	33,256 (6339)	**0.013**	−3870 [−6913 to −826]
sICAM1 (ng/mL)	97 (35)	88 (38)	0.25	10 [−7 to 27]
sVCAM1 (ng/mL)*	328 (110)	281 (86)	**0.04**	47 [2 to 92]
sVEGFR1 (pg/mL)	203 (162)	229 (129)	0.44	−26 [−94 to 41]
sVEGFR2 (pg/mL)*	10,369 (3468)	13,627 (4423)	**0.0009**	−3258 [−5135 to −1381]
sVEGFR3 (pg/mL)	2271 (2521.8)	2359 (1818)	0.86	−88 [−1094 to 919]
TRAIL (pg/mL)	49 (68)	68 (38)	0.14	−19 [−44 to 6.2]
VEGFA (pg/mL)	676 (488)	485 (378)	0.06	191.2 [−9 to 392]
VEGFC (pg/mL)	2141 (914)	2058 (1143)	0.75	82.6 [−430 to 595]
VEGFD (pg/mL)*	754 (463)	549 (334)	**0.03**	205 [18 to 391]

Abbreviations: AT1R: angiotensin II type1- receptor antibody; CCL: chemokine (C-C motif) ligand; CXCL4: C-X-C motif ligand 4; ETAR: anti-endothelin -1 type A receptor antibody; FGF: fibroblast growth factor; IL: interleukin; MMP: matrix metalloproteinase; PDGF: platelet derived growth factor; sICAM: soluble intercellular adhesion molecule; sVCAM: soluble vascular cell adhesion molecule; sVEGFR: soluble vascular endothelial growth factor receptor; TRAIL: TNF-related apoptosis-inducing ligand.

* Indicating factors discriminative between SSc-PH and SSc-noPH.

Bold values indicate statistically significant values with a *p* of <0.05.

### RF model

**Figure 2.**
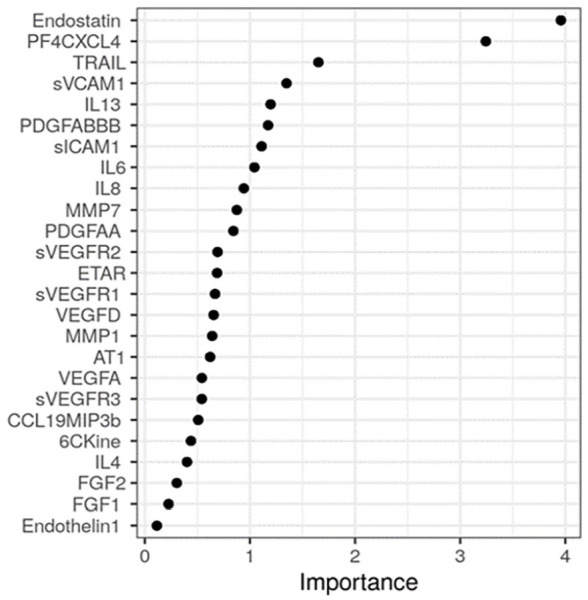
Random forest model, reflecting the importance of the measured factors discriminative between SSc-PH and SSc-noPH across 500 decision trees. Factors with the highest importance are the most predictive. Abbreviations: AT1: angiotensin II type1- receptor antibody; CCL: chemokine (C-C motif) ligand; PF4CXCL4: platelet factor4/ C-X-C motif ligand 4; ETAR: anti-endothelin -1 type A receptor antibody; FGF: fibroblast growth factor; IL: interleukin; MMP: matrix metalloproteinase; PDGF: platelet derived growth factor; sICAM: soluble intercellular adhesion molecule; sVCAM: soluble vascular cell adhesion molecule; sVEGFR: soluble vascular endothelial growth factor receptor; TRAIL: TNF-related apoptosis-inducing ligand.

**Figure 3.**
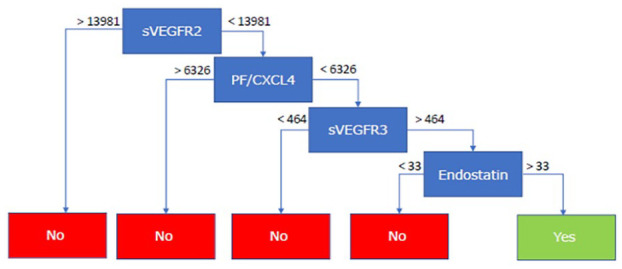
Example of 1 of the 500 decision trees, incorporated in the random forest model, in which YES represents SSc-PH and NO represents SSc-noPH. sVEGFR2/3 in pg/mL, CXCL4 in ng/mL, Endostatin in ng/mL. Abbreviations: CXCL4: C-X-C motif ligand4; sVEGFR: soluble vascular endothelial growth factor receptor.

The RF model, incorporating all 26 soluble serum markers, identified Endostatin and PF4/CXCL4 as the most discriminative factors between SSc-PH and SSc-noPH (Figure 2). An example of one of the 500 incorporated decision trees in the model is displayed in Figure 3. The AUC of the model with the best fit was 0.92, indicating a good discriminative performance of this model (Supplemental Figure A).

## Discussion

In this study, we explored the discriminating potential of NCM and 26 soluble serum factors between SSc-PH and SSc-noPH. We found statistically significant differences in 6 of these markers. Furthermore, RF analysis identified Endostatin and CXCL4 as the most important and potential disease classifying soluble factors for SSc-PH.

In our study, we found no differences between SSc-PH and SSc-noPH in both qualitative and quantitative nailfold characteristics. Also, looking at the SSc-PAH subgroup specifically, no statistical significant differences in quantitative or qualitative nailfold parameters were found compared to patients with other types of associated PH, or SSc patients without PH. Our findings are in contrast with the findings summarized in a recent systematic review that concluded that a lower capillary density and/or an active/late SSc pattern was associated with SSc-PAH.^35^ The capillary density of the SSc-PH patients we describe is similar to the SSc-PAH patients described in the included cross-sectional studies in this review. However, the density we describe for SSc-noPH patients is lower. The differences between these studies and our results may be explained by a difference in sample size, as we included more SSc-noPH patients. Our opinion is that our observations regarding the SSc-noPH NCM properties are representative for these patients, as the capillary density for SSc-noPH patients is comparable to the density described in another recent and larger cross-sectional study, including 11 SSc-PAH and 123 SSc-noPH patients.^36^ This latter study confirms our findings with regard to the lack of association between SSc-PAH and lower capillary density or combined active/late pattern. Based on our results, the evidence to further explore the value of adding NCM to screenings algorithms PAH in SSc is currently insufficient.

Previous research on the vascular receptor-autoantibodies antiAT1R and anti-ETAR in SSc-PAH is contradictive. One large study in 298 SSc patients, of which 13.8% had PAH, revealed that patients with high vascular autoantibody levels had an increased relative risk of PAH.^23^ However, another large study including 67 SSc-PAH patients, 217 SSc-noPH, and PH patients of other etiologies, elevated vascular-autoantibodies were identified in SSc-PAH, and CTD-PAH compared with PH of other etiologies, but the antibody levels were overall equally high or even slightly higher in patients with SSc that did not develop PAH.^24^ In our study, vascular autoantibody titer does not differentiate between SSc-PH and SSc-noPH, which is in line with the latter study. Therefore, our results currently do not add evidence to explore additional value of adding these vascular autoantibodies to screening algorithms for PAH in SSc.

We developed a preliminary RF model, incorporating all measured soluble serum markers in this study. The advantage of an RF model is that it ultimately offers a relative feature importance that allows to select the most contributing features for classification of patients easily. The model can be quite challenging to interpret, in comparison to a single decision tree; a model like this is based on multiple decision trees. The RF model we describe specified Endostatin and CXCL4 as the most discriminative soluble factors for PH in SSc. Our results indicate that the additional value of these factors above other factors already incorporated in current screening algorithms should be further explored.

Interestingly, both Endostatin and CXCL4 can be linked to inhibition of the endothelial cell function. Endostatin has been found to inhibit pulmonary artery endothelial cell proliferation and migration, and promotes endothelial cell apoptosis, important features involved in PAH pathophysiology.^37^ These findings were confirmed in a study including two largely unselected cohorts, showing that endostatin levels were elevated in both patients with SSc and mixed connective tissue disease (MCTD).^30^ Furthermore, in this study elevated endostatin was associated with both scleroderma renal crisis and PAH in SSc. Endostatin was recently described by Bauer et al.,^38^ as part of a proteomic-biomarker signature that could discriminate SSc patients with and without PAH. In accordance with this, we found that circulating endostatin was upregulated both in SSc-PAH and other types of SSc-PH, compared with no-PH, indicating this to be a promising marker. CXCL4 is a chemokine with potent antiangiogenic properties which is secreted by megakaryocytes, activated platelets, and plasmacytoid dendritic cells.^26^ Previous proteome-wide analysis revealed the role of CXCL4 as a biomarker for development of PAH in SSc.^27^ In contrast to the latter, we found CXCL4 to be significantly lower in SSc-PH patients. A possible explanation for this difference could be a difference in patient characteristics and measurement in serum versus plasma, as well as variations in pre-analytical sample preparations, such as collection, processing, and storage, which can be critical since platelet activation during clotting may result in CXCL4 release.^39^ Despite the fact that CXCL4 has high discriminative value for PH in our RF model, downregulation of this factor in SSc-PH patients is in contrast with the current literature on CXCL4 and further research is necessary to decide if CXCL4 is a promising marker to be further explored.

Next to Endostatin, we also observed sVCAM and VEGF-D to be significantly elevated in SSc-PH. Both are markers of vascular injury and were described earlier to be elevated in LcSSc PAH patients.^32^ Our research group recently described several soluble markers including VEGFD that could possibly discriminate pathophysiological different phenotypes in SSc.^33^ Possibly angiogenesis, or a disturbance of this process in SSc-PH, could also be reflected by the ratio between VEGFD and VEGFR2, which could be an interesting observation to explore in future studies. In contrast to what we expected, PDGF was significantly decreased in SSc-PH. It is hypothesized that hypoxia from progressive PAH may accelerate vascular injury by stimulating increased ET-1, VEGF, PDGF, and endothelial apoptosis.^40^

To date, this is the only cross-sectional study in which NCM parameters, vascular autoantibodies, and cytokine profile have been studied in SSc patients with and without PH of different etiologies. Our study has several strengths, including the extensive clinical data, collected in a tertiary referral center for both SSc and PH, detailed assessments for NCM in accordance with international standards^14^ making it possible to compare our results with previous reported findings, and the important confirmation of the presence of P(A)H by RHC.

Furthermore, we employed a novel machine-based forest classification method aimed at identifying the most discriminative potential biomarkers for PAH in SSc.

With regard to limitations, it is important to emphasize that our results should be interpreted with caution, considering the relatively small size of this exploratory single-center study, and its cross-sectional design. Furthermore, we did not match patients for age, disease duration, SSc subtype, or presence of ILD, reflecting real-life differences between SSc patients with and without associated PH. In daily clinical practice, SSc patients without PH also use specific vasodilator therapy in the case of DUs, and both SSc patients with and without PH can have various indications for the use of immunosuppressive therapy, reflecting the heterogeneity of SSc. The use of vasodilator and immunosuppressive therapy, and the presence of ILD or DUs may have had an important influence on both the NCM findings and the expression levels of the measured autoantibodies and cytokines.^22^ Exploring whether the differentially expressed cytokine levels are identifiable before PH diagnosis in SSc, and the changes in response to both immunosuppressive and vasoactive therapy should be a next topic on the research agenda.

Summarizing, our study shows that SSc patients with and without PH can potentially be differentiated based on the serum cytokine expression profile in which elevated Endostatin seems to be the most promising discriminative factor. Furthermore, we show the potential for a machine learning−based RF model to be of aid in selecting the most discriminative markers.

To support our data, an adequately powered prospective cohort study, including patients with newly diagnosed PAH, is needed to address the important question of whether these biomarkers can indeed be used for risk stratification for PAH in patients with SSc.

## Supplemental Material

sj-pdf-1-jso-10.1177_23971983231175213 – Supplemental material for Nailfold capillaroscopy and candidate-biomarker levels in systemic sclerosis-associated pulmonary hypertension: A cross-sectional study Click here for additional data file. Supplemental material, sj-pdf-1-jso-10.1177_23971983231175213 for Nailfold capillaroscopy and candidate-biomarker levels in systemic sclerosis-associated pulmonary hypertension: A cross-sectional study by Jacqueline MJ Lemmers, Arjan PM van Caam, Brigit Kersten, Cornelia HM van den Ende, Hanneke Knaapen, Arie PJ van Dijk, Wanda Hagmolen of ten Have, Frank HJ van den Hoogen, Hans Koenen, Sander I van Leuven, Wynand Alkema, Ruben L Smeets and Madelon C Vonk in Journal of Scleroderma and Related Disorders

## References

[1] Denton CP Khanna D. Systemic sclerosis. Lancet 2017; 390(10103): 1685–1699. 28413064 10.1016/S0140-6736(17)30933-9

[2] Hachulla E Launay D Mouthon L , et al. Is pulmonary arterial hypertension really a late complication of systemic sclerosis? Chest 2009; 136(5): 1211–1219. 19429720 10.1378/chest.08-3042

[3] Vonk MC Vandecasteele E van Dijk AP. Pulmonary hypertension in connective tissue diseases, new evidence and challenges. Eur J Clin Invest 2021; 51(4): e13453. 10.1111/eci.13453 PMC7988614 33216992

[4] Wigley FM Lima JA Mayes M , et al. The prevalence of undiagnosed pulmonary arterial hypertension in subjects with connective tissue disease at the secondary health care level of community-based rheumatologists (the UNCOVER study). Arthritis Rheum 2005; 52(7): 2125–2132. 15986394 10.1002/art.21131

[5] Avouac J Airò P Meune C , et al. Prevalence of pulmonary hypertension in systemic sclerosis in European Caucasians and metaanalysis of 5 studies. J Rheumatol 2010; 37(11): 2290–2298. 20810505 10.3899/jrheum.100245

[6] Tyndall AJ Bannert B Vonk M , et al. Causes and risk factors for death in systemic sclerosis: a study from the EULAR Scleroderma Trials and Research (EUSTAR) database. Ann Rheum Dis 2010; 69(10): 1809–1815. 20551155 10.1136/ard.2009.114264

[7] Denton CP. Advances in pathogenesis and treatment of systemic sclerosis. Clin Med 2015; 15(Suppl. 6): s58–s63. 10.7861/clinmedicine.15-6-s58 26634684

[8] Lefèvre G Dauchet L Hachulla E , et al. Survival and prognostic factors in systemic sclerosis-associated pulmonary hypertension: a systematic review and meta-analysis. Arthritis Rheum 2013; 65(9): 2412–2423. 23740572 10.1002/art.38029

[9] Humbert M Yaici A de Groote P , et al. Screening for pulmonary arterial hypertension in patients with systemic sclerosis: clinical characteristics at diagnosis and long-term survival. Arthritis Rheum 2011; 63(11): 3522–3530. 21769843 10.1002/art.30541

[10] Coghlan JG Denton CP Grünig E , et al. Evidence-based detection of pulmonary arterial hypertension in systemic sclerosis: the DETECT study. Ann Rheum Dis 2014; 73(7): 1340–1349. 23687283 10.1136/annrheumdis-2013-203301 PMC4078756

[11] Galie N Humbert M Vachiery JL , et al. 2015 ESC/ERS guidelines for the diagnosis and treatment of pulmonary hypertension: the joint task force for the diagnosis and treatment of pulmonary hypertension of the European Society of Cardiology (ESC) and the European Respiratory Society (ERS): endorsed by: Association for European Paediatric and Congenital Cardiology (AEPC), International Society for Heart and Lung Transplantation (ISHLT). Eur Heart J 2016; 37(1): 67–119. 26320113 10.1093/eurheartj/ehv317

[12] Launay D Sanges S Sobanski V. Time for precision medicine in systemic sclerosis-associated pulmonary arterial hypertension. Eur Respir J 2021; 57(6): 2100205. 34168056 10.1183/13993003.00205-2021

[13] Avouac J Lepri G Smith V , et al. Sequential nailfold videocapillaroscopy examinations have responsiveness to detect organ progression in systemic sclerosis. Semin Arthritis Rheum 2017; 47(1): 86–94. 28291582 10.1016/j.semarthrit.2017.02.006

[14] Smith V Herrick AL Ingegnoli F , et al. Standardisation of nailfold capillaroscopy for the assessment of patients with Raynaud’s phenomenon and systemic sclerosis. Autoimmun Rev 2020; 19(3): 102458. 31927087 10.1016/j.autrev.2020.102458

[15] Ong YY Nikoloutsopoulos T Bond CP , et al. Decreased nailfold capillary density in limited scleroderma with pulmonary hypertension. Asian Pac J Allergy Immunol 1998; 16(2–3): 81–86. 9876945

[16] Hofstee HM Vonk Noordegraaf A Voskuyl AE , et al. Nailfold capillary density is associated with the presence and severity of pulmonary arterial hypertension in systemic sclerosis. Ann Rheum Dis 2009; 68(2): 191–195. 18375538 10.1136/ard.2007.087353

[17] Riccieri V Vasile M Iannace N , et al. Systemic sclerosis patients with and without pulmonary arterial hypertension: a nailfold capillaroscopy study. Rheumatology 2013; 52(8): 1525–1528. 23671125 10.1093/rheumatology/ket168

[18] Marino Claverie L Knobel E Takashima L , et al. Organ involvement in Argentinian systemic sclerosis patients with “late” pattern as compared to patients with “early/active” pattern by nailfold capillaroscopy. Clin Rheumatol 2013; 32(6): 839–843. 23417347 10.1007/s10067-013-2204-8

[19] Corrado A Correale M Mansueto N , et al. Nailfold capillaroscopic changes in patients with idiopathic pulmonary arterial hypertension and systemic sclerosis-related pulmonary arterial hypertension. Microvasc Res 2017; 114: 46–51. 28619664 10.1016/j.mvr.2017.06.005

[20] Ohtsuka T Hasegawa A Nakano A , et al. Nailfold capillary abnormality and pulmonary hypertension in systemic sclerosis. Int J Dermatol 1997; 36(2): 116–122. 9109008 10.1046/j.1365-4362.1997.00088.x

[21] Minopoulou I Theodorakopoulou M Boutou A , et al. Nailfold capillaroscopy in systemic sclerosis patients with and without pulmonary arterial hypertension: a systematic review and meta-analysis. J Clin Med 2021; 10(7): 1528. 33917407 10.3390/jcm10071528 PMC8038744

[22] Lemmers JMJ Velauthapillai A van Herwaarden N , et al. Change of the microvascularization in systemic sclerosis, a matter of air. Best Pract Res Clin Rheumatol 2021; 35(3): 101683. 33814313 10.1016/j.berh.2021.101683

[23] Riemekasten G Philippe A Näther M , et al. Involvement of functional autoantibodies against vascular receptors in systemic sclerosis. Ann Rheum Dis 2011; 70(3): 530–536. 21081526 10.1136/ard.2010.135772

[24] Becker MO Kill A Kutsche M , et al. Vascular receptor autoantibodies in pulmonary arterial hypertension associated with systemic sclerosis. Am J Respir Crit Care Med 2014; 190(7): 808–817. 25181620 10.1164/rccm.201403-0442OC

[25] Pignone A Scaletti C Matucci-Cerinic M , et al. Anti-endothelial cell antibodies in systemic sclerosis: significant association with vascular involvement and alveolo-capillary impairment. Clin Exp Rheumatol 1998; 16(5): 527–532. 9779298

[26] Odler B Foris V Gungl A , et al. Biomarkers for pulmonary vascular remodeling in systemic sclerosis: a pathophysiological approach. Front Physiol 2018; 9: 587. 29971007 10.3389/fphys.2018.00587 PMC6018494

[27] Van Bon L Affandi AJ Broen J , et al. Proteome-wide analysis and CXCL4 as a biomarker in systemic sclerosis. New Engl J Med 2014; 370(5): 433–443. 24350901 10.1056/NEJMoa1114576 PMC4040466

[28] Liu H Yang E Lu X , et al. Serum levels of tumor necrosis factor-related apoptosis-inducing ligand correlate with the severity of pulmonary hypertension. Pulm Pharmacol Ther 2015; 33: 39–46. 26086178 10.1016/j.pupt.2015.06.002

[29] Hummers LK Hall A Wigley FM , et al. Abnormalities in the regulators of angiogenesis in patients with scleroderma. J Rheumatol 2009; 36(3): 576–582. 19228661 10.3899/jrheum.080516 PMC4020014

[30] Reiseter S Molberg O Gunnarsson R , et al. Associations between circulating endostatin levels and vascular organ damage in systemic sclerosis and mixed connective tissue disease: an observational study. Arthritis Res Ther 2015; 17: 231. 26315510 10.1186/s13075-015-0756-5 PMC4551562

[31] Hoffmann-Vold AM Hesselstrand R Fretheim H , et al. CCL21 as a potential serum biomarker for pulmonary arterial hypertension in systemic sclerosis. Arthritis Rheumatol 2018; 70: 1644–1653. 29687634 10.1002/art.40534

[32] Pendergrass SA Hayes E Farina G , et al. Limited systemic sclerosis patients with pulmonary arterial hypertension show biomarkers of inflammation and vascular injury. PLoS ONE 2010; 5(8): e12106. 10.1371/journal.pone.0012106 PMC2923145 20808962

[33] Smeets RL Kersten BE Joosten I , et al. Diagnostic profiles for precision medicine in systemic sclerosis; stepping forward from single biomarkers towards pathophysiological panels. Autoimmun Rev 2020; 19(5): 102515. 32173517 10.1016/j.autrev.2020.102515

[34] Van den Hoogen F Khanna D Fransen J , et al. 2013 classification criteria for systemic sclerosis: an American college of rheumatology/European league against rheumatism collaborative initiative. Ann Rheum Dis 2013; 72(11): 1747–1755. 24092682 10.1136/annrheumdis-2013-204424

[35] Smith V Vanhaecke A Vandecasteele E , et al. Nailfold videocapillaroscopy in systemic sclerosis-related pulmonary arterial hypertension: a systematic literature review. J Rheumatol 2020; 47(6): 888–895. 31416927 10.3899/jrheum.190296

[36] Guillén-Del-Castillo A Simeón-Aznar CP Callejas-Moraga EL , et al. Quantitative videocapillaroscopy correlates with functional respiratory parameters: a clue for vasculopathy as a pathogenic mechanism for lung injury in systemic sclerosis. Arthritis Res Ther 2018; 20(1): 281. 30567570 10.1186/s13075-018-1775-9 PMC6299957

[37] Goyanes AM Moldobaeva A Marimoutou M , et al. Functional impact of human genetic variants of COL18A1/endostatin on pulmonary endothelium. Am J Respir Cell Mol Biol 2020; 62(4): 524–534. 31922883 10.1165/rcmb.2019-0056OC PMC7110972

[38] Bauer Y de Bernard S Hickey P , et al. Identifying early pulmonary arterial hypertension biomarkers in systemic sclerosis: machine learning on proteomics from the DETECT cohort. Eur Respir J 2021; 57(6): 2002591. 33334933 10.1183/13993003.02591-2020 PMC8276065

[39] Lande R Lee EY Palazzo R , et al. CXCL4 assembles DNA into liquid crystalline complexes to amplify TLR9-mediated interferon-α production in systemic sclerosis. Nat Commun 2019; 10(1): 1731. 31043596 10.1038/s41467-019-09683-z PMC6494823

[40] Faller DV. Endothelial cell responses to hypoxic stress. Clin Exp Pharmacol Physiol 1999; 26(1): 74–84. 10027074 10.1046/j.1440-1681.1999.02992.x

