# Supplementary material for: Nailfold capillaroscopy and candidate-biomarker levels in systemic sclerosis-associated pulmonary hypertension: A cross-sectional study
Source: J Scleroderma Relat Disord. 2023 May 22;8(3):221–30. doi: 10.1177/23971983231175213 (PMC10515989; doi:10.1177/23971983231175213)
Supplement: sj-pdf-1-jso-10.1177_23971983231175213 – Supplemental material for Nailfold capillaroscopy and candidate-biomarker levels in systemic sclerosis-associated pulmonary hypertension: A cross-sectional study [file sj-pdf-1-jso-10.1177_23971983231175213.pdf]

Supplemental file

Supplemental table A: Detailed clinical information on SSc-PH group, with data on SSc-PAH and SSc-PH of other etiologies presented separately.

	SSc-PAH (n=21)	SSc-PH-Other (n=19)
**Age, years (median, IQR)**	71 (67.5-76.5)	73 (71-77)
**Female (n,%)**	16 (76.2%)	14 (73.7%)
**SSc duration years (median, IQR)**	12.6 (7.4-18.8)	5.9 (3.4-15.8)
**SSc Subtype**		
**LcSSc (n,%)**	18 (85.7%)	14 (73.7%)
**DcSSc (n, %)**	3 (14.3%)	4 (21.1%)
**SSc sine Scleroderma**	0	1 (5.3%)
**ILD (n%)**	10 (47.6)	16 (84.2%)
**ILD duration, years (median, IQR)**	5.29 (1.21-9.31)	5.71 (3.58-11.88)
**PH duration years (median, IQR)**	3.42 (1.38-8.71)	4 (1.5-6.0)
**Digital ulcers (n, %)**	13 (61.9%)	8 (42.1%)
**Telangiectasias (n,%)**	20 (95.2%)	19 (100%)
**NYHA class (n, %)**		
**I**	0 (0%)	2 (10.5%)
**II**	5 (23.8%)	5 (26.3%)
**III**	15 (71.4%)	6 (31.6%)
**IV**	1 (4.8%)	6 (31.6%)
**Not reported**	0	0
**Creat umol/L (median, IQR)**	97 (82-110)	105 (76-123)
**Urate mmol/L (median, IQR)**	0.39 (0.28-0.47) n=18	0.37 (0.34-0.52) n=18
**Nt-proBNP pg/ml (median, IQR)**	540 (235-1050)	650 (390-2900)
**ANA positive (n, %)**	19 (90.5%) n=19	17 (89.5%) n=17
**ACA**	13 (61.9%)	7 (36.8%)
**Anti Topoisomerase I**	0	4 (21.1%)
**antiRNAIII**	1 (4.8%)	0
**antiU1RNP**	1 (4.8%)	0
**Anti-SSA**	3 (15%)	4 (10%)
**AntPM-Scl**	1 (5%)	1 (5%)
**Anti-histon**	1 (5%)	1 (5%)
**VC % pred**	84% (73-103) n=19	84,5% (69,5-100%) (n= 16)
**DLCO% pred**	39 (31.5-47.5) (n=18)	39.5% (28.25-41) (n=16)
**6min WT meters (median, IQR)**	384.5 (292- 440) n=18	300 (235-390) n=13
**Comedication use (n, %)**		
**Immunosuppression**	8 (38.1%)	13 (68.4%)
**MMF**	0	9 (47.4%)
**Prednisone**	6 (28.6%)	9 (47.4%)
**HCQ**	1 (4.8%)	0
**Methotrexate**	1 (4.8%)	0

**cyclo**	1 (4.8%)	0
**other**	1 (4.8%)	0
**PH medication**		
**noPH medication**	0	6 (31.6 %)
**ERA**	19 (90.5%)	8 (42.1%)
**PDE5**	17 (80.1%)	9 (47.3%)
**PGI2**	7 (33.3%)	3 (15.8%)
**riociguat**	1 (4.8%)	2 (10.5%)
**Mono therapy**	4 (19%)	6 (31.6%)
**Duo therapy**	11 (52.4%)	5 (26.3)
**Tripple therapy**	6 (28.6%)	2 (10.5)

Supplemental Table A: Detailed clinical information on SSc-PH group, with data on SSc-PAH and SSc-PH of other etiologies presented separately. Values presented as mean Median IQR or n%.
Abbreviations: ANA; antinuclear antibodies , ACA; Anti-centromere antibodies, antiRNAIII; Anti-RNA polymerase III antibodies, antiU1RNP; Anti-U1RNP antibodies, Cyclo; Cyclophosphamide, DcSSc; diffuse cutaneous Systemic Sclerosis, DLCO%pred; Diffusion capacity for Carbon monoxide as percentage of predicted, ERA; Endothelin receptor antagonists, HCQ; Hydroxychloroquine, ILD; interstitial Lung Disease, LcSSc; Limited cutaneous Systemic Sclerosis, MMF; Mycophenolate Mofetil, NTproBNP; N-terminal pro hormone of brain natriuretic peptide, NYHA; New York Heart Association functional Class, PDE5i; Phosphodiesterase-5 inhibitors, PGI2; Prostacyclin analogues, PH; Pulmonary Hypertension, SSc; Systemic Sclerosis, VC%pred; Vital Capacity as percentage of predicted 6minWT; 6 minute walking test.

Supplemental Table B; Nailfold capillary characteristics compared between subgroups, evaluated by ANOVA (not corrected for Age, SSc disease duration or DLCO% predicted).

	**SSc-noPH (n=39)**	**SSc-PAH (n=21)**	**SSc-PH other (n=18)**	**p value (ANOVA)**
CAPILLARIES (MEAN, SD)				
Density/mm	5.3 (1.9)	4.8 (1.5)	5.2 (1.3)	0.57
Mean number of fingers with density ≤3/mm	1.8 (2.1)	2.5 (1.9)	2.2 (1.9)	0.41
Giant capillaries/mm (>50um)	0.24 (0.3)	0.19 (0.3)	0.11 (0.1)	0.17
Abnormal morphology/mm	0.99 (0.53)	1.2 (0.38)	1.2 (0.6)	0.15
Digits with hemorrhages present	1.6 (± 1.5)	1.9 (2.2)	1.8 (1.6)	0.79
**overall Pattern (n,%)**				0.29
Early SSc	3 (7.7%)	0	0	
Active SSc	8 (20.5%)	2 (9.5%)	2 (11.1%)	
Late SSc	25 (64.1%)	19 (90.5%)	15 (83.3%)	
Non-scleroderma (normal/a specific)	3 (7.7%)	0	1 (5.6%)	

Supplemental Table B: Nailfold Capillaroscopy Characteristics.
Between group comparison *for continuous variables* with ANOVA*, for categorical variables by pearson Chi. Quantitative characteristics mean SD, Qualitative (NCM Pattern (n%).*

Supplemental Table C

Soluble serum-factors	Lower limits of detection
CCL19 (pg/ml)	8,70
CCL21 (6Ckine) (pg/ml)	91,25
CXCL4 (ng/ml)	0,04
Endothelin1 (pg/ml)	8,86
Endostatin (ng/ml)	0,00
FGF1 (pg/ml)	13,71
FGF2 (pg/ml)	51,89
IL4 (pg/ml)	0,64
IL6 (pg/ml)	0,62
IL8 (pg/ml)	0,68
IL13 (pg/ml)	36,47
MMP1 (pg/ml)	27,43
MMP7 (pg/ml)	548,46
PDGF-AA (pg/ml)	12,92
PDGF AB-BB (pg/ml)	5935,04
sICAM1 (ng/ml)	0,09
sVCAM1 (ng/ml)	0,12
sVEGFR1 (pg/ml)	13,55
sVEGFR2 (pg/ml)	137,68
sVEGFR3 (pg/ml)	411,75
TRAIL (pg/ml)	9,64
VEGFA (pg/ml)	12,90
VEGFC (pg/ml)	23,74
VEGFD (pg/ml)	22,72

Supplemental Figure A: ROC curve

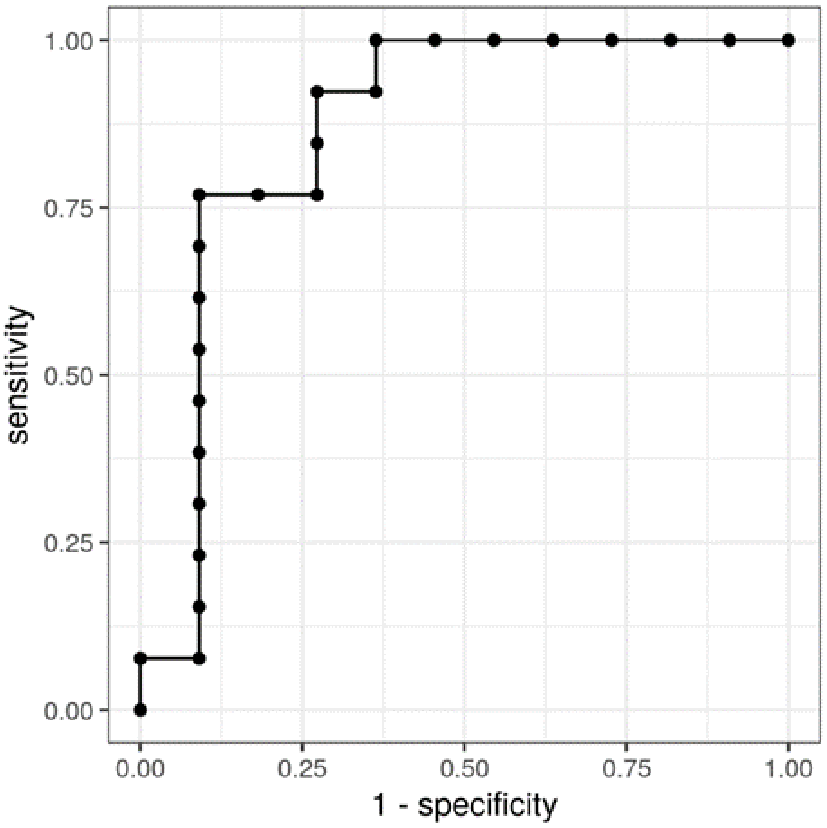

Figure A: ROC Receiver operating Curve, reflecting performance of in total 500 decision trees generated by the Random Forest model, in which the area under the curve (AUC) is 0.92.
